# dbCNV: deleteriousness-based model to predict pathogenicity of copy number variations

**DOI:** 10.1186/s12864-023-09225-4

**Published:** 2023-03-20

**Authors:** Kangqi Lv, Dayang Chen, Dan Xiong, Huamei Tang, Tong Ou, Lijuan Kan, Xiuming Zhang

**Affiliations:** 1 Xinxiang Medical University, 453003 Xinxiang, China; 2 Medical Laboratory of the Third Affiliated Hospital of Shenzhen University, No. 47 of Youyi Road, 518001 Shenzhen City, Guangdong Province China

**Keywords:** Copy number variation, Pathogenicity, XGBoost, Machine learning

## Abstract

**Background:**

Copy number variation (CNV) is a type of structural variation, which is a gain or loss event with abnormal changes in copy number. Methods to predict the pathogenicity of CNVs are required to realize the relationship between these variants and clinical phenotypes. ClassifyCNV, X-CNV, StrVCTVRE, etc. have been trained to predict the pathogenicity of CNVs, but few studies have been reported based on the deleterious significance of features.

**Results:**

From single nucleotide polymorphism (SNP), gene and region dimensions, we collected 79 informative features that quantitatively describe the characteristics of CNV, such as CNV length, the number of protein genes, the number of three prime untranslated region. Then, according to the deleterious significance, we formulated quantitative methods for features, which fall into two categories: the first is variable type, including maximum, minimum and mean; the second is attribute type, which is measured by numerical sum. We used Gradient Boosted Trees (GBT) algorithm to construct dbCNV, which can be used to predict pathogenicity for five-tier classification and binary classification of CNVs. We demonstrated that the distribution of most feature values was consistent with the deleterious significance. The five-tier classification model accuracy for 0.85 and 0.79 in loss and gain CNVs, which proved that it has high discrimination power in predicting the pathogenicity of five-tier classification CNVs. The binary model achieved area under curve (AUC) values of 0.96 and 0.81 in the validation set, respectively, in gain and loss CNVs.

**Conclusion:**

The performance of the dbCNV suggest that functional deleteriousness-based model of CNV is a promising approach to support the classification prediction and to further understand the pathogenic mechanism.

**Supplementary Information:**

The online version contains supplementary material available at 10.1186/s12864-023-09225-4.

## Background

Copy number variation (CNV) is a type of structural variation, which is a gain or loss event with abnormal changes in copy number involving DNA fragments, typically longer than 50 bp [1]. Based on a high-resolution CNV map constructed using publicly available data, CNVs cover 4.8–9.5% of the genome [2]. CNVs involve coding genes and important functional elements, and can affect gene dosage and gene expression, with significant implications for phenotypic variation and disease [3, 4]. CNVs have been shown to increase the risk of autism spectrum disorders, developmental delays and other neurodevelopmental disorders [5–7]. Therefore, it is necessary to explore the pathogenicity of CNV. However, the pathogenicity classification and phenotypic analysis of CNVs are time-consuming and difficult tasks, requiring experienced experts to analyze and integrate genomic information. Concurrent with the technical advances in CNV identification, the method of machine learning provides convenient and fast conditions for predicting the pathogenicity of CNVs. Most importantly, it can realize the comparison of the results in different laboratories and promote the consistency of test results.

Approaches of machine learning for predicting CNV pathogenicity can be divided into two categories: (1) implementing heuristics based on reference medical guidelines the American College of Medical Genetics and Genomics (ACMG) and the Clinical Genome Resource (ClinGen) [8], the results of each item are evaluated according to the content of the guidelines, and then the standard five-tier classification (pathogenic, likely pathogenic, uncertain significance, benign, likely benign) is used to judge the pathogenicity of CNV, such as ClassifyCNV [9] and AutoCNV [10]. The implementation of the guidelines relies heavily on individual competence. Clinical and genetic expertise are required, the lack of which affects the accuracy of the prediction and thus limits its application for large-scale DNA sequencing data. The advent of these tools has addressed this issue. ClassifyCNV is a command line program that discriminates multi-class CNV and is not user-friendly to clinical applications. AutoCNV is a standard-based semiautomated tool for CNV interpretation that is previously available in a web version to encourage user submissions. However, the bulk upload capability of AutoCNV should be developed for CNV interpretation. (2) Supervised learning method predicted the pathogenicity of CNVs, which is a method to construct model and perform automatic classification based on extensive functional annotation and training datasets. Although functional annotation of CNVs has been considered in genome-wide annotation and the calculation of feature values has been done by counting or taking the mean, no statistics based on the deleterious significance of features have been presented. Several tools have been developed, but few of these tools predicted the pathogenicity of the five-tier classification of CNVs, such as X-CNV [11], ISV [12], StrVCTVRE [13] and TADA [14]. X-CNV is an approach to integrate diverse human genome information toward a quantitative measure of pathogenicity of CNVs on the whole genome-scale. To quantitatively measure the relationship between CNV and pathogenicity, X-CNV developed a meta-voting prediction (MVP) score. The area under the curve (AUC) values of MVP score in discriminating benign and pathogenic CNVs were 0.76 and 0.83. ISV achieved more than 98% prediction accuracy on both loss and gain variants when discriminating between benign and pathogenic CNVs, while also allowing CNVs being assigned “uncertain” significance in predictions. StrVCTVRE is a model that focuses on exon boundaries to distinguish benign rare SVs from pathogenic rare SVs. StrVCTVRE performed well of accuracy (91%) on clinical SVs and achieved AUC of 0.83. TADA is a tool that focuses on functional regions to distinguish between pathogenic and non-pathogenic variants. The authors trained a classifier utilizing features describing coding regions, enhancers, TAD boundaries, and CTCF binding site. The AUC values for the pathogenic and benign ClinVar sets were 0.89 and 0.84, which were improvement over the performance X-CNV and StrVCTVRE.

**Fig. 1:**
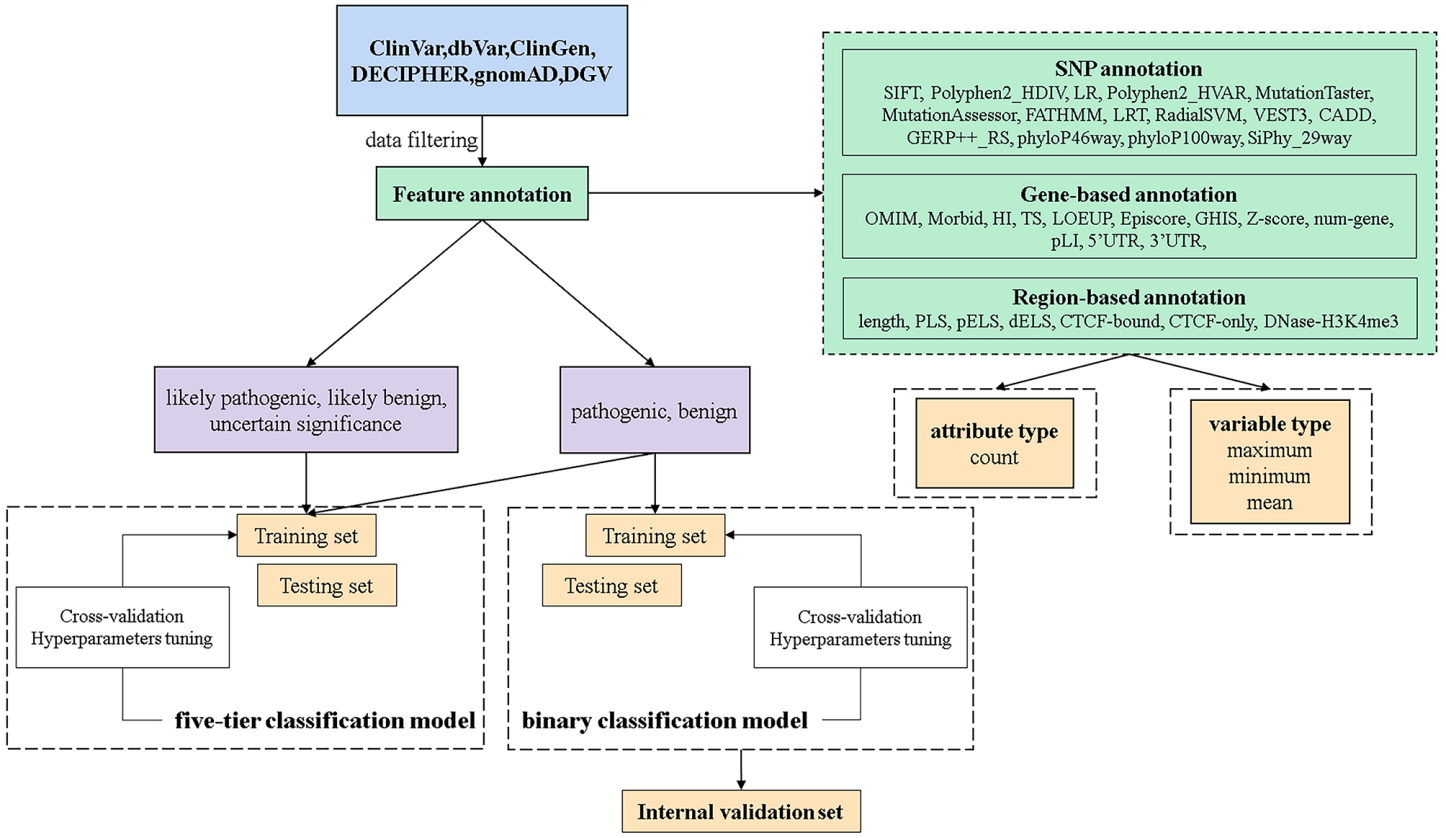
Workflow of dbCNV training and validation. The model was trained based on the XGBoost algorithm using 79 features of CNVs. The five-tier classification model and binary classification model were constructed based on CNVs from multiple databases. Features were quantified by variable type and attribute type methods

In this manuscript, we constructed dbCNV to predict the pathogenicity of CNVs based on the deleterious significance of features. The quantitative evaluation of features was based on their pathogenicity levels in CNVs of different classifications. As shown in Fig. 1, we described the feature annotation process, the generation of train and test sets from multiple databases, the model construction and the validation process. Finally, a better AUC was achieved on five-tier classification model, and a higher accuracy was obtained in the clinical validation of binary classification model.

## Result

### Distribution of feature values in different classification CNVs

**Fig. 2:**
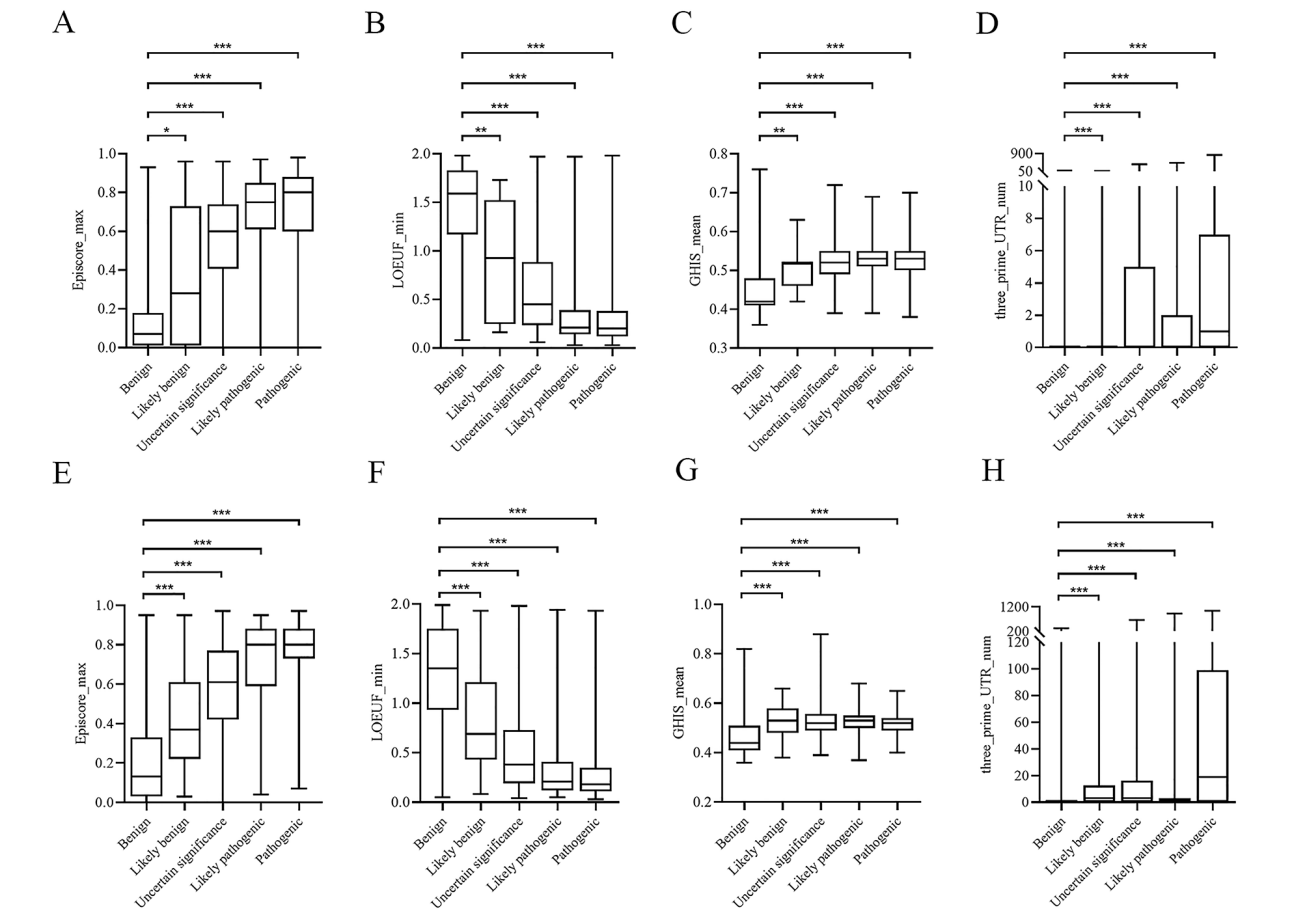
Distribution of Episcore_max (**A**), LOEUF_min (**B**), GHIS_mean (**C**) and three_prime_UTR_num (**D**) in loss CNVs. Distribution of Episcore_max (**E**), LOEUF_min (**F**), GHIS_mean (**G**) and three_prime_UTR_num (**H**) in gain CNVs. (*, p < 0.05; **, p < 0.01; ***, p < 0.001)

In feature annotation, we made quantification based on the deleterious significant of the features, and finally divided feature values annotation into four types: maximum, minimum, mean and count. To verify whether this quantification method was reasonable, we selected one feature description from each of the four annotation types. We selected Episcore_max, LOEUP_min, GHIS_mean, and three_prim_UTR_num for statistics. Since features were typically not regularly distributed, the nonparametric Mann-Whitney U test was used to evaluate their significance. Using the benign group as a reference, it was confirmed whether the feature values of other pathogenic groups were significantly different from those of the benign group. Ultimately, the other pathogenic groups of these four features were all statistically different from the benign group. (p-value < 0.05) (Fig. 2). Episcore_max and LOEUP_min showed a significant progressive trend conformed to the score of deleterious significance. Although GHIS_mean and three_prim_UTR_num did not have significant propensity to progress, the values of the features can distinguish between benign and pathogenic. We also used the Kruskal-Wallis H Test and post hoc Pairwise Comparisons on these four features, the results showed that the histogram judgment of the overall distribution shape of the feature values in each group was not consistent, and the difference was statistically significant (p < 0.05). Post hoc pairwise comparisons using Bonferroni corrected significance levels found statistically significant differences between benign and pathogenic groups. However, not all differences in the likely benign, likely pathogenic, and uncertain significance groups were statistically significant (see supplement Table 3).

### Genomic element features of CNVs in database

**Fig. 3:**
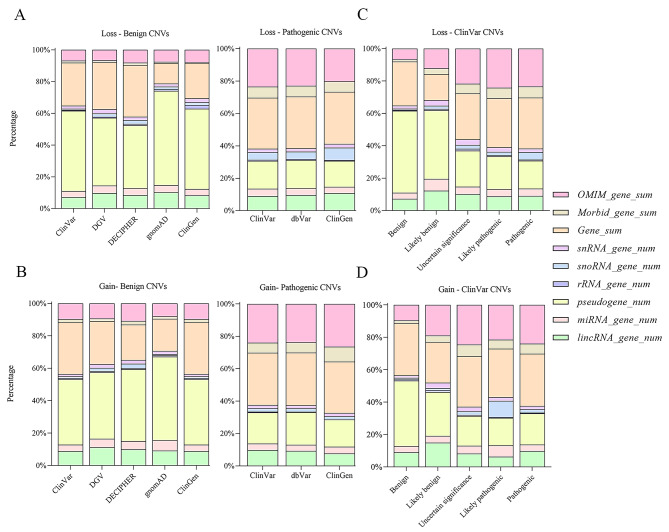
The proportion of genomic element features in different databases. **A** The proportion of genomic element features of benign and pathogenic loss CNVs in different databases. **B** The proportion of genomic element features of benign and pathogenic gain CNVs in different databases. **C** The proportion of genomic element features of loss CNVs in ClinVar database. **D** The proportion of genomic element features of gain CNVs in ClinVar database

We classified CNVs according to the percentage of various genomic elements located within the variation region. The genomic elements consist of pseudogene, OMIM gene, Morbid gene, RNA gene and protein-coding gene. In benign CNVs, the number of pseudogenes from different databases accounted for 40–60% of genomic elements, which was the most in gnomAD and the least in DECIPHER. On the contrary, the involvement of pseudogenes was slightly in contrast to benign CNVs, in which pathogenic CNVs accounted for less than 17%. OMIM and Morbid genes in benign CNVs accounted for 8% of the genomic elements, whereas them accounted for 30% of the pathogenic CNVs. However, same trends emerged for RNA gene features in benign and pathogenic CNVs, with percentages ranging from 17 to 20% in both (Fig. 3A-B). The differences were confirmed in the ClinVar database, where the percentages of pseudogenes, OMIM and Morbid genes in benign and pathogenic CNVs were significantly different (Fig. 3C-D). The number of RNA genes and protein-coding genes were roughly equal in percentage in the five-tier classification. Only the benign CNVs from the gnomAD database had the smallest number of protein-coding genes, which is 13% of all various genomic elements. Similar trends were observed in the loss and gain CNVs separately.

### Important features of the model

**Fig. 4:**
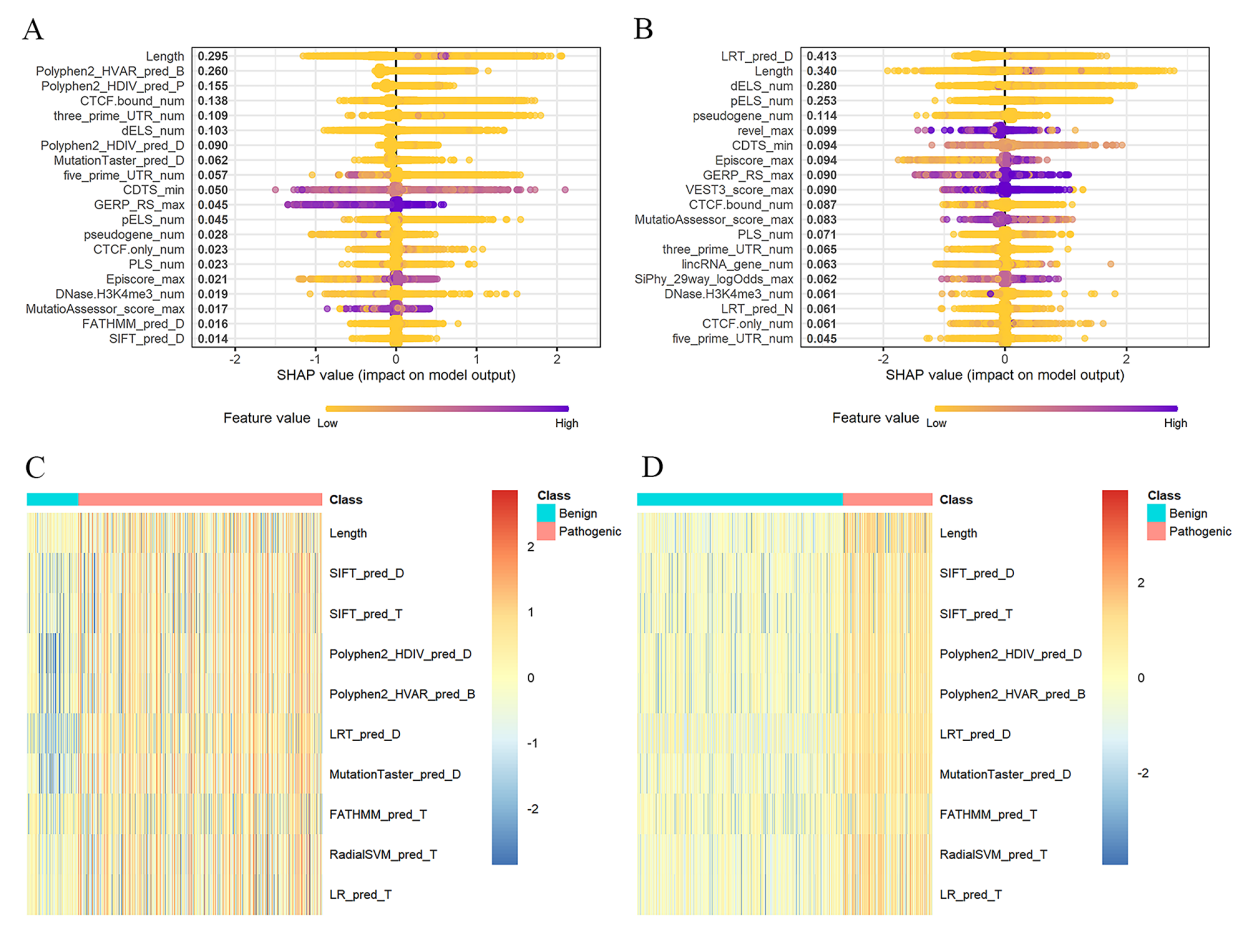
SHAP summary plot of the top 20 features of the model. The x axis measures the impact on the model output (right positive, left negative). Taking the feature of length as an example, purple points are on the right. This means prediction effect will be positive when CNVs have a long level of length. SHAP summary plot of the top 20 features of the five-tier classification model for loss **(A)** and gain **(B)** CNVs. Differential expression analysis for pathogenic and benign CNVs features. ​The classes of pathogenic and benign CNVs were shown separately in red and blue. Heat map of the differential analysis in loss **(C)** and gain **(D)** CNVs

To enhance the explain ability of models, we depicted the SHapley Additive exPlanations (SHAP) summary plot of the top twenty features in five-tier classification model for gain and loss (Fig. 4A-B). Feature values were indicated by a spectrum with purple representing the highest value. According to the constructed model, the higher the SHAP value of a feature, the more likely it was to classify the pathogenicity of CNVs. Features were ranked according to the sum of absolute SHAP values over all CNVs, and the distribution of the impact of each feature on the full model output was shown. There was a difference between the SHAP values of the loss and gain CNVs features (Fig. 4A-B). In SHAP value plot of the loss CNVs, length, Polyphen2_HVAR_pred and Polyphen2_HDIV_pred were the most important features for distinguishing the pathogenicity of CNVs. However, in the gain CNVs, LRT_pred, length and dELS were the most important features.

To confirm the efficacy of all annotated features in discriminating pathogenicity of CNVs, we performed an analysis of all features. We performed differential expression analysis only for benign and pathogenic CNV features in the loss and gain CNVs separately. To eliminate the influence of extreme values, we normalized all the feature values by a log10 transform. The top ten features from the differential analysis were selected, and CNVs with all feature values of 0 were removed. Finally, 2,231 benign CNVs and 965 pathogenic CNVs were shown in the loss heat map, and 2,048 benign CNVs and 9,764 pathogenic CNVs in the gain heat map (Fig. 4C-D). As shown in figure, benign and pathogenic CNVs have significant difference of features expression.

### Performances of five-tier classification model and binary classification model in test sets

**Fig. 5:**
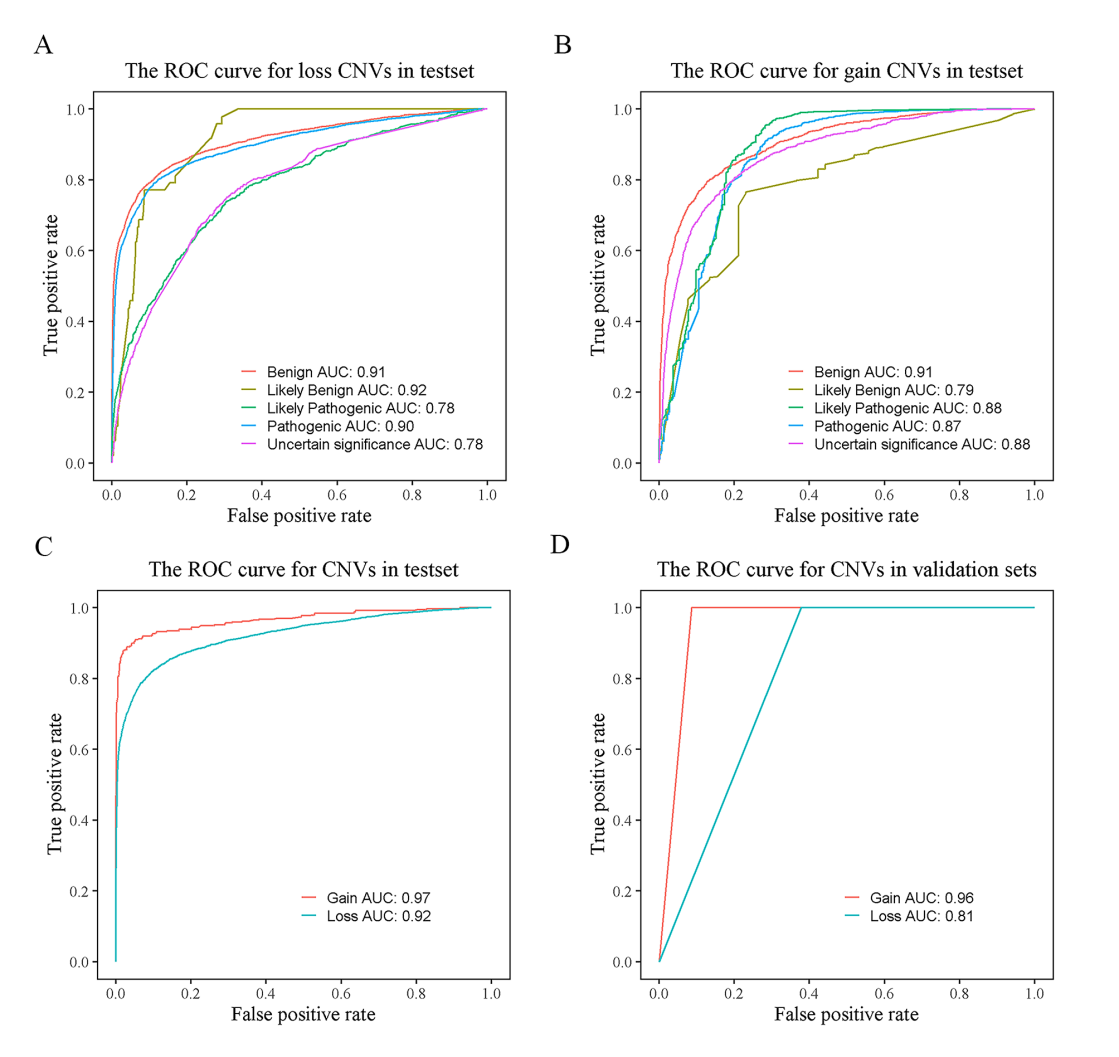
ROC curves for the test and validation sets. **A** AUC values for the test set of loss CNVs. **B** AUC values for the test set of gain CNVs. **C** AUC values for the test set of binary classification model. **D** AUC values for the validation set of binary classification model

The performances of the dbCNV in predicting the pathogenicity of CNVs were measured by the accuracy and AUC values. In discriminating among different pathogenicity CNVs in test set of the five-tier classification model, the accuracy of the loss (0.85) was higher than the gain CNVs (0.79). We found that benign (0.91) had the same AUC in both types of CNVs separately. The loss CNVs yielded higher AUC values in pathogenic (0.90) and likely benign (0.92) than the gain CNVs. Moreover, likely pathogenic and uncertain significance had higher AUC (i.e., likely pathogenic 0.88, uncertain significance 0.88) in the gain CNVs (Fig. 5A-B). Furthermore, the binary classification model yielded a higher AUC for gain CNVs (0.97) compared with loss CNVs (0.92) (Fig. 5C), and accuracy were 0.90 and 0.97 in loss and gain CNVs.

### Validation of manually classified datasets in the model

To assess whether the model performed well on an independent database, we evaluated our method on a set of CNVs determined by two clinicians (see Supplementary Table 2). These CNVs were obtained from our internal dataset. The AUC values of gain CNVs (0.96) in the validation set was higher than loss CNVs (0.81) (Fig. 5D).

### Performances comparison of dbCNV with other models

Our five-tier classification model outperformed ClassifyCNV in three performance metrics, i.e., balance accuracy, sensitivity, and specificity. For example, the accuracy of gain CNVs for the pathogenic of the model was 0.90, improving 80% over ClassifyCNV (Table 1). The ClassifyCNV yielded the highest sensitivity of uncertain significance CNVs (i.e., loss 0.95, gain 0.98). For the comparison of binary classification models, our model outperformed StrVCTVRE. dbCNV can accurately classify pathogenic CNVs with a sensitivity of 1 for duplication and deletion (Table 2).

**Table 1:** Model performance of dbCNV and ClassifyCNV on ClinVar set

metrics	dbCNV												ClassifyCNV											
	loss						gain						loss						gain					
	P	LP	VUS	LB	B		P	LP	VUS	LB	B		P	LP	VUS	LB	B		P	LP	VUS	LB	B	
Balance accuracy	**0.69**	**0.59**	0.60	0.50	**0.74**		**0.90**	**0.82**	**0.89**	**0.85**	**0.90**		0.53	0.45	**0.67**	0.50	0.17		0.50	0.50	0.52	0.50	0.59	
Sensitivity	**0.68**	**0.18**	0.20	0	**0.85**		**0.80**	**0.65**	0.83	**0.69**	**0.95**		0.06	0.16	**0.95**	0	0.33		0.01	0.002	**0.98**	0	0.19	
Specificity	0.69	**0.99**	**0.99**	1	**0.64**		0.99	0.99	0.95	0.99	0.83		**0.99**	0.74	0.38	1	0.01		0.99	0.99	**0.05**	**1**	**0.99**	

**Table 2:** Model performance of dbCNV and StrVCTVRE on ClinVar set

metrics	dbCNV			StrVCTVRE	
	loss	gain		loss	gain
Balance accuracy	**0.80**	**0.95**		0.79	0.84
Sensitivity	**1**	**1**		0.78	0.91
Specificity	0.60	**0.91**		**0.80**	0.77
F1 score	0.67	0.83		**0.87**	**0.92**
precision	0.50	0.71		**0.99**	**0.94**

## Discussion

Although several models have been developed to predict the pathogenicity of CNVs, specifically in binary classification model, the calculation of feature values and the distribution of feature values in different pathogenicity still need to be studied. For example, in X-CNV, the deleterious prediction scores were calculated for CNVs by dividing the sum of the scores of the variants falling within the CNV region by the covered CNV length, and the deleterious significance of the features to the disease is not considered. We aimed to construct an automated method for quantifying and annotating scores based on the deleterious significance of features to predict the pathogenicity of CNVs. The method required only basic information about the location and type of a CNV, i.e., the genomic coordinates and whether genomic region had been loss or gain. The features quantification of CNVs can be divided into two categories: (1) variable type features, (2) attribute type features. The model created a curated benchmark CNV list by combining publicly available CNV resources to generate a comprehensive list of CNV pathogenicity annotated by features. We constructed dbCNV to predict pathogenicity of the five-tier classification and binary classification CNVs. dbCNV has a unique ability to integrate feature information into quantitative annotation of CNV pathogenicity based on deleterious significance. This particular method was proved to be suitable by analyzing the distribution of feature values, the proportion of genomic elements, and SHAP analysis. Furthermore, dbCNV achieved better performances compared to a state-of-the-art technique on datasets, in which loss typically appeared to be more challenging to classify than gain CNVs. Finally, for the classification of pathogenic CNVs in the verification set, our binary classification model got perfect results.

In order to observe whether the values of feature were reasonable, we drew boxplots of the features according to the pathogenicity of CNVs. The range of the distribution of all feature values changed as the degree of pathogenicity increased, and the variable type changed more significantly than the attribute type. In addition, we found that the feature values partially overlapped in the two groups: benign and likely benign, pathogenic and likely pathogenic. Moreover, most of the uncertain significance overlapped with likely benign and likely pathogenic. However, there was a slight overlap in the feature values of benign and pathogenic CNVs. It indicated that our feature quantification method worked well at discriminating between benign and pathogenic CNVs. In ClinVar database, CNVs were classified by manual review, which included genetic data, evidence of allele or genotype consistent with the practice guidelines, and the support of other literature, etc. [15]. Therefore, the judgment of pathogenicity of CNVs was supported by clinical phenotypes, and the distribution of feature annotation values in different classifications correspond to the results of manual classification. Moreover, the nonparametric Mann-Whitney U test was used to evaluate the significance of feature values for different pathogenicity CNVs. Virtually all the features with p value lower than 0.001. However, the range of LOEUF_min values almost overlapped between likely pathogenic and pathogenic indicated that the feature failed to classify the two classes CNVs. In contrast, we found that the degrees of progressive of variable type features were more significant than that of attribute type features in different pathogenicity CNVs. We suspected that the methods used to quantify attribute type features did not fully demonstrate the deleterious significance.

Graphical interpretation of CNV feature values from different databases, we strongly recommend computing and plotting cumulative bar diagram. We have previously shown that the proportion of genomic element features can be used to estimate the pathogenicity of CNV. For pathogenic CNVs, similar trends were observed in ClinVar, dbVar and ClinGen databases. However, for benign CNVs, the proportion of features were different across five databases. Based on the above, we suspected that the databases had consistent classification criteria for pathogenic CNVs, but the classification of benign CNVs was controversial. Therefore, data from multiple databases should be taken into account when the model was trained to reduce the bias caused by differences. In ClinVar database, although the proportions of genomic element features significantly differed in the performances of classification of benign and pathogenic CNVs, the proportions data were challenging for the classification of likely benign, likely pathogenic and uncertain significance. Therefore, a comprehensive evaluation of CNVs was needed to improve the accuracy of model predictions.

**Fig. 6:**
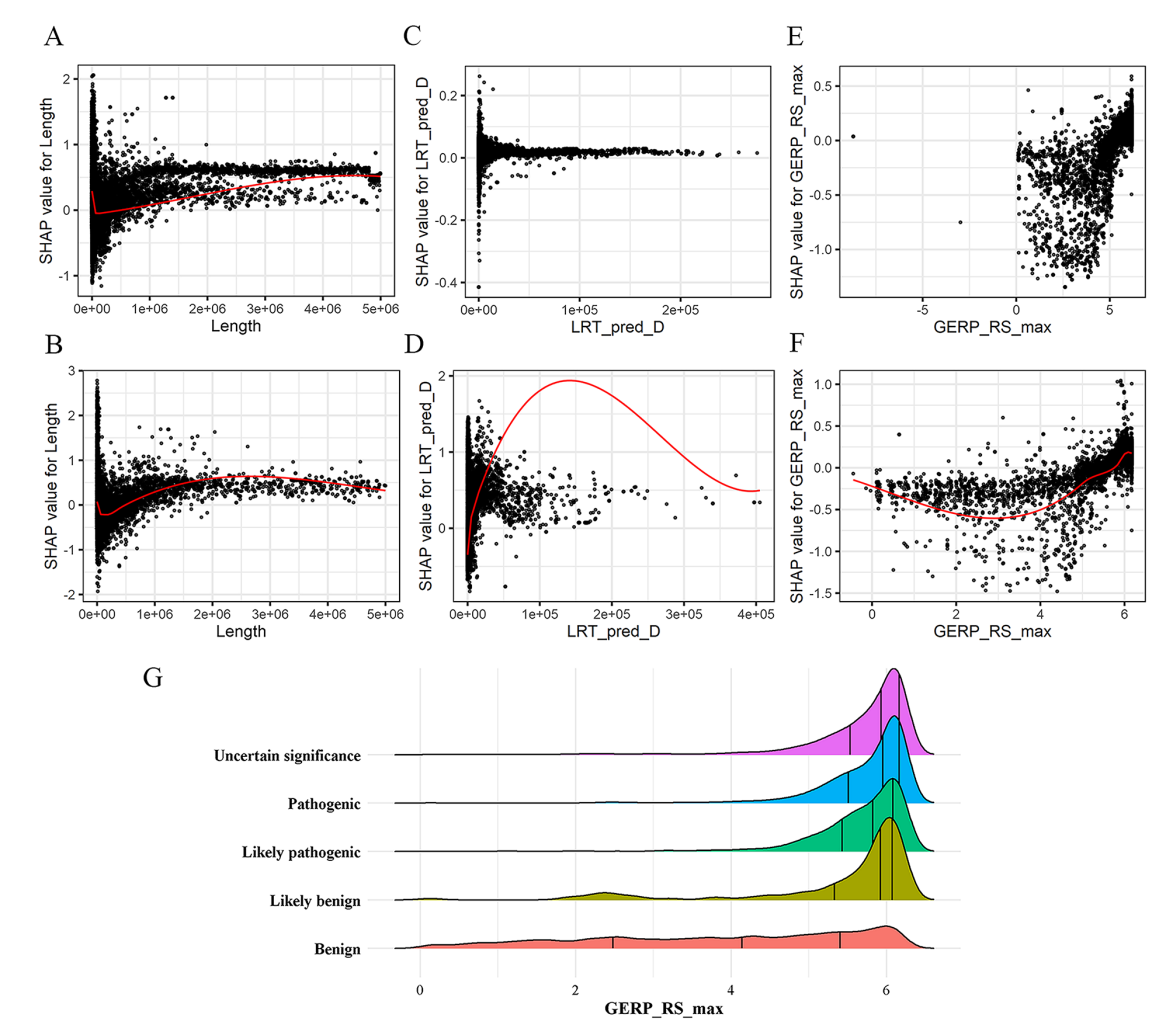
The SHAP dependence plot of the features. SHAP dependence plot for length **(A)**, LRT_pred_D **(C)** and GERP_RS_max **(E)** values of the loss CNVs. SHAP dependence plot for length **(B)**, LRT_pred_D **(D)** and GERP_RS_max **(F)** values of the gain CNVs. **G** Distribution of GERP_RS_max values for five-tier classification CNVs. The quartile values are indicated with a vertical bar

We have shown that the SHAP values can interpret the inner workings of model. Despite SHAP values were calculated theoretically by observing the effect each feature contributes to the final prediction, the concordance between feature values and their SHAP values were not perfect. In other words, the magnitudes of the feature values were not consistent with the influence of the feature on pathogenicity. This means that the same values of the features in different CNVs will have different impacts on the final predictions. We visualized the SHAP values for individual features, which can be used to understand how individual features affect the output of the prediction model. However, the influence of the extreme values of some attribute type features caused most of the values to cluster. For example, in the length SHAP dependency plot, the feature values range from 0 to 1 × 10^6^ bp and the distribution range of SHAP values was wide (Fig. 6A-B). When the feature value was greater than 1 × 10^6^ bp, the SHAP values were greater than 0, which shown a positive effect on the pathogenicity model prediction. Therefore, we suggested that the longer length of CNV, the more likely it was to cause pathogenic. This was consistent with the result that the length of pathogenic CNVs were larger than that of benign CNVs in X-CNV. In LRT_pred_D SHAP dependency plot of gain CNVs, most of the feature values were in the range of 0-50000 and the SHAP values were greater than 0, which had a positive effect on the model prediction (Fig. 6D). As seen from the SHAP dependency plot of GERP_RS_max in Fig. 6E-F, the SHAP values were smaller than 0 when the feature values were smaller than 5 and larger than 0 when the feature values were larger. We found that virtually all the GERP_RS_max values less than 5 were benign CNVs, but the other pathogenic feature values were not distinctive (Fig. 6G). Based on the above findings, we suspected that GERP_RS_max value of 5 was the threshold to discriminate benign CNVs.

We also found that the top three features contributing to the model were different in the SHAP analysis of gain and loss CNVs, separately. In the SHAP value plot of loss CNVs, the most important feature to the model was the length of CNV. But in gain CNVs, the most important feature was LRT_pred_D, which counted the number of LRT categorical predictions as “Deleterious” in CNV. When the LRT_pred_D values were greater than 50,000, the SHAP values were around 0 for the loss CNVs and greater than 0 for the gain CNVs (Fig. 6C-D). This suggested that features in different types of CNVs had different effects on predicting pathogenicity. According to the Society of Obstetricians and Gynaecologists of Canada (SOGC)-Canadian College of Medical Geneticists (CCMG) guideline, to minimize the reporting of uncertain findings, it is recommended that variants of unknown significance (VOUS) smaller than 500 Kb loss or 1 Mb gain not be routinely reported [16]. It follows that the threshold for gain CNVs was larger than that for loss. Therefore, the feature of length weas more important in predicting pathogenicity of CNVs in the loss than in gain CNVs. Study had shown that gain CNVs less than 1 Mb in length of uncertain significance have limited effect on improving diagnostic yield [17]. Additionally, the features that contributed most to the model in this study were different from those in other studies. In TADA, the most relevant features were the predicted haploinsufficiency of the closest gene and the HI Log Odds score. In ISV, the number of morbid genes turned out to be one of the most important features together with regulatory elements in both gain and loss CNVs. The inconsistency between the results of our study and those of other studies may be due to the differences in the methods used to quantify features and the fact that some features were not considered by other studies.

Despite dbCNV performs well as a machine learning model on both test and validation sets, there are limitations that need to be addressed in the future. dbCNV had two main shortcomings: (1) the features annotation is not comprehensive. We did not consider functional elements and regional features, such as: TAD boundary [18], constrained coding region (CCR) [19], enhancer and especially CNV breakpoint [20]. The assessment of the pathogenicity of breakpoints in genes was discussed in accordance with the ACMG guidelines, but similar to other tools did not consider the effect of breakpoints on the pathogenicity of CNV. (2) The results of binary classification model were not perfect in our internal dataset. This may be due to the fact that the validation set was classified as pathogenic and non-pathogenic in manual judgment by two clinicians, but dbCNV classified the validation set as pathogenic and benign. However, the actual pathogenicity of CNVs predicted as benign may be likely benign, likely pathogenic and uncertain significance. This error led to unsatisfactory results.

## Conclusion

In summary, dbCNV is able to predict the pathogenicity of CNVs based on the deleterious significance of the feature annotations, which can further explore the pathogenicity mechanism of CNVs. We believe that the predictive power of dbCNV has the potential to assist in clinical diagnosis.

## Methods

### Datasets

To annotate CNVs with relevant features, we downloaded CNV data from several publicly available databases, including ClinVar [21], dbVar [22], ClinGen [23], Database of Genomic Variants (DGV) [1], DECIPHER [24], gnomAD [25]. In ClinVar database, CNVs were retained if they fulfilled all the following requirements: (1) type of copy number gain, copy number loss, deletion or duplication; (2) clinical significance of pathogenic, likely pathogenic, benign, likely benign or uncertain significance; (3) review status as “criteria provided, multiple submitters, no conflicts”, “criteria provided, single submitter”, “reviewed by expert panel” or “practice guideline”. dbVar database was restricted to copy number gain, copy number loss, deletion or duplication variants, with labelled as “Pathogenic”, “Likely pathogenic” or “pathogenic/likely pathogenic”. ClinGen database was restricted to dosage sensitive regions. A haploinsufficiency/triplosensitivity score 3 of the regions to be dosage sensitive for a loss/gain associated with clinical phenotype was regarded as pathogenic CNVs. A haploinsufficiency/triplosensitivity score 40 was regarded as benign CNVs. DGV database was collected from DGV Gold Standard Variants. CNVs with total number of samples tested less than 2000 were filtered. In DECIPHER database, the filtering criteria for CNV were: “observations” > 0 and frequency > 1%. gnomAD variants were restricted to those with SVTYPE equal to “DEL” or “DUP”, FILTER equal to “PASS”, allele frequency (AF) > 1% and allele number (AN) > 2000. Due to selection, we expect pathogenic variants to occur at low frequencies, while more frequent variants should be benign or less harmful.

In order to merge CNVs from different databases into a non-redundant dataset, the data were processed as follows: (1) the length of CNVs ranging from 50 bp to 5 × 10^6^ bp were retained; (2) CNVs with a reciprocal overlap greater or equal to 70% and conflicting pathogenicity were removed; (3) the CNVs had multiple pathogenicity significance were removed. After the above procedures, the following CNVs were filtered: 3,472 CNVs from ClinVar, 4,444 CNVs from dbVar, 42 CNVs from DECIPHER, 315 CNVs from DGV, 11 CNVs from gnomAD, 38 CNVs from ClinGen.

### CNV feature annotation

From single nucleotide polymorphism (SNP), gene and region dimensions, we collected important features of CNV, and then these features derived some other relevant features from different ways of expression quantification. Finally, 79 features were collected for annotation (see Supplementary Table 1). The features quantification of CNVs can be divided into two categories: Variable type features and Attribute type features.

### Variable type features

Variable type features included various functional deleteriousness scores from ANNOVAR, a software of annotations for variant sites. Since the scores were calculated at locus-level or gene-level, we then calculated these scores for CNVs by comparing the size of the scores with deleterious significance of the variants falling within the CNV regions. Those features could be grouped into four aggregation methods: (1) when the deleterious significance of the features is that the higher scores are more deleterious, the maximum of scores as the feature value. Such features include Probability of Loss-of-function intolerant (pLI) score [26], Rare Exome Variant Ensemble Learner (REVEL) score [27], Genome Evolutionary Rate Profiling ++ (GERP++_RS) score [28], Episcore [29], etc. The pLI score is the probability that a given gene falls into the Haploinsufficient category, and therefore is extremely intolerant of loss-of-function variation. Genes with high pLI scores (pLI ≥ 0.9) are extremely LoF intolerant, whereby genes with low pLI scores (pLI ≤ 0.1) are LoF tolerant. REVEL score is predicting the deleteriousness of each nucleotide change in the genome. The score for an individual missense variant can range from 0 to 1, with higher scores reflecting greater likelihood disease-causing. Genomic Evolutionary Rate Profiling (GERP) is a method for producing position-specific estimates of evolutionary constraint using maximum likelihood evolutionary rate estimation. Constraint intensity at each individual alignment position is quantified in terms of a “rejected substitutions” (RS) score. Episcore is a computational method (Episcore) to predict haploinsufficiency leveraging epigenomic data from a broad range of tissue and cell types by machine learning methods. (2) Lower scores are more deleterious, these score of features were calculated the minimum as feature value, includes Loss-of-function Observed / Expected Upper bound Fraction (LOEUF) score [30], Sort intolerated from tolerated (SIFT) score [31], Functional Analysis Through Hidden Markov Models (FATHMM) score, etc. LOEUF score range from 0 (most depleted/evolutionarily constrained) to 9 (not depleted/constrained). Each gene was also analyzed by using the pLI score. SIFT score is a normalized probability of observing the new amino acid at that position, and ranges from 0 to 1. A value of between 0 and 0.05 is predicted to affect protein function. FATHMM score predicts the functional consequences of cancer-associated amino acid substitutions using a model weighted for inherited disease mutations. (3) Calculated the mean score of variants as feature value if no biological significant: genome-wide haploinsufficiency score (GHIS), which score evaluates the haploinsufficiency [32]. (4) Others. The length of CNV was calculated by the end position minus start position.

### Attribute type features

The following features associated with CNV were counted to assess: (1) the number of genes or regions reciprocal overlap in queried CNV: protein-coding genes, UTR regions, non-coding RNA genes, pseudogenes, and downloaded from Ensembl; (2) the number of categorical predictions of deleteriousness scores that overlap in queried CNV: SIFT-pred, LRT_pred and PolyPhen2_pred, etc. Likelihood ratio test (LRT) score ranges from 0 to 1 and a larger score signifies that the codon is more constrained or a nonsynonymous SNP is more likely to be deleterious [33]. PolyPhen2 (Polymorphism Phenotyping v2) is a tool which predicts possible impact of an amino acid substitution on the structure and function of a human protein using straightforward physical and comparative considerations [34]. The categorical predictions are measured by “D: Deleterious”, “B: benign”, “T: Tolerated”, etc. (3) The number of the Registry of candidate cis-Regulatory Elements (cCREs) reciprocal overlap in queried CNV: CTCF-bound, CTCF-only, dELS, DNase-H3K4me3, pELS, PLS. cCREs are the subset of representative DNase hypersensitivity sites (rDHSs) supported by either histone modifications (H3K4me3 and H3K27ac) or CTCF-binding data. Many uses of cCREs are based on the regulatory role associated with their biochemical signatures [35]; (4) the number of haploinsufficiency (HI) / triplosensitivity (TS) genes with score of 0/1/2/3/40/NA (available from ClinGen); (5) the number of OMIM/Morbid genes (available from Online Mendelian Inheritance in Man) in CNV [36].

As a quality control, the coordinates of features regions were recalculated based on GRCh37/hg19 by using the UCSC genome browser liftOver tool [37], and only those that remained the same length, without splitting into discontinuous genomic intervals after this process were retained. In the features annotating process, we found apart of feature values were missing value, which might affect the accuracy of model construction. We used median to fill them.

### CNV model constructed

Ultimately, we collected 89,104 CNVs from six several publicly available databases for model training and testing. We constructed five-tier classification and binary classification model, and loss and gain types of CNVs were considered separately. The datasets of dbCNV were randomly split into training and test cohorts with a 7:3 ratio. Multi-class classification model was trained on 57,523 benign, 339 likely benign, 7,678 uncertain significance, 19,650 pathogenic and 3,913 likely pathogenic CNVs. Binary classification model was trained by 57,523 benign and 19,650 pathogenic CNVs. dbCNV was constructed with two categories features based on Gradient Boosted Trees (GBT) classification algorithm, a stochastic gradient boosting classification model (XGBoost). To determine the optimal performance, the hyperparameters of model were optimized by using the Bayesian optimization tool Optuna in 10-fold-cross validation setting. The hyperparameters eta, gamma, max_depth, min_child_weight, subsample and nrounds were tuned to control overfitting and enhance the better performance. For the other hyperparameters, we used the default values. The hyperparameters that gave the best performance were chosen for the training of the final XGBoost model.

### Validation

The binary classification model was validated using CNVs from our internal database. To ensure that the validation dataset did not reciprocal overlapped, CNVs with overlap of at least 70% and conflicting pathogenicity annotations were filtered. For any pair of CNVs that share a reciprocal overlap of at least 90% of their respective lengths based on genomic coordinates, we selected the shorter of the two. Because the length was limited during the features annotation process, we only selected length of CNVs ranging from 50 bp to 5 × 10^6^ bp. After the filtering described above, the CNVs of validation dataset were identified by two clinicians and classified as pathogenic or benign. The sensitivity, specificity, Receiver operating characteristic (ROC) analysis and AUC value were calculated to evaluate the discrimination ability.

### Comparative analysis of other models

Since dbCNV can be used to predict pathogenicity for five-tier classification and binary classification of CNVs, ClassifyCNV and StrVCTVRE were chosen for performance comparison, respectively. For performance comparison, the models were run in Windows environment. The default parameters were used for the models. CNVs used for model comparison were obtained from ClinVar database. The performance metrics to evaluate the five-tier model are balance accuracy, sensitivity, and specificity, and the binary model are balance accuracy, sensitivity, and specificity, F1 score and precision.

## Electronic supplementary material

Below is the link to the electronic supplementary material.

**Additional file 1: Supplementary Table 1.** List of features used by dbCNV. **Supplementary Table 2.** Internal dataset. **Supplementary Table 3.** P values for pairwise comparisons of feature values.

## Data Availability

dbCNV can be accessed at: https://github.com/lllllv-1/dbCNV. The following public data resources and tools were listed: ClinVar(https://ftp.ncbi.nlm.nih.gov/pub/dbVar/sandbox/dbvarhub/hg19/). dbVar (https://ftp.ncbi.nlm.nih.gov/pub/dbVar/sandbox/sv_datasets/nonredundant/). ClinGen (https://ftp.clinicalgenome.org/ClinGen_region_curation_list_GRCh37.tsv). Database of Genomic Variants (http://dgv.tcag.ca/dgv/docs/DGV.GS.March2016.50%.GainLossSep.Final.hg19.gff3). DECIPHER (https://www.deciphergenomics.org/files/downloads/population_cnv_grch37.txt.gz). gnomAD(https://storage.googleapis.com/gcp-public-data--gnomad/papers/2019-sv/gnomad_v2.1_sv.controls_only.sites.bed.gz). CDTS (http://www.hli-opendata.com/noncoding/). ANNOVAR (https://annovar.openbioinformatics.org/en/latest/user-guide/download/). Ensemble (https://ftp.ensembl.org/pub/grch37/release-87/gff3/homo_sapiens/Homo_sapiens.GRCh37.87.chr.gff3.gz). SCREEN (Search Candidate cis-Regulatory Elements by ENCODE, https://downloads.wenglab.org/V3/GRCh38-cCREs.bed). UCSC genome browser liftOver tool (https://genome.ucsc.edu/cgi-bin/hgLiftOver). R (v3.6.0, https://www.r-project.org/).

## List of abbreviations

CNV: copy number variation
SNP: single nucleotide polymorphism
AUC: achieved area under curve
ACMG: American College of Medical Genetics and Genomics
ClinGen: Clinical Genome Resource
DGV: Database of Genomic Variants
ROC: Receiver operating characteristic
GBT: Gradient Boosted Trees
MVP: Meta-voting prediction
SHAP: SHapley Additive exPlanations
CCR: constrained coding region
AN: allele number
pLI: Probability of Loss-of-function intolerant
REVEL: Rare Exome Variant Ensemble Learner
GERP ++_RS: Genome Evolutionary Rate Profiling + + rejected substitutions
LOEUF: Loss-of-function Observed / Expected Upper bound Fraction
SIFT: Sort intolerated from tolerated
FATHMM: Functional Analysis Through Hidden Markov Models
GHIS: genome-wide haploinsufficiency score
LRT: Likelihood ratio test
PolyPhen2: Polymorphism Phenotyping v2
